# Characterization of the gut microbiota in polycystic ovary syndrome with dyslipidemia

**DOI:** 10.1186/s12866-024-03329-x

**Published:** 2024-05-17

**Authors:** Tianjin Yang, Guanjian Li, Yuping Xu, Xiaojin He, Bing Song, Yunxia Cao

**Affiliations:** 1 Reproductive Medicine Center, Department of Obstetrics and Gynecology, the First Affiliated Hospital of Anhui Medical University, Hefei, 230032 China; 2 NHC Key Laboratory of Study on Abnormal Gametes and Reproductive Tract, Anhui Medical University, Hefei, 230032 China; 3 Ministry of Education Key Laboratory of Population Health Across Life Cycle, Anhui Medical University, Hefei, 230032 China; 4 Anhui Province Key Laboratory of Reproductive Health and Genetics, Hefei, 230032 China; 5 Biopreservation and Artificial Organs, Anhui Provincial Engineering Research Center, Anhui Medical University, Hefei, 230032 China; 6 Reproductive Medicine Center, Shanghai General Hospital, Shanghai Jiao Tong University School of Medicine, Shanghai, China

**Keywords:** Polycystic ovary syndrome, Dyslipidemia, Gut microbiota

## Abstract

**Background:**

Polycystic ovary syndrome (PCOS) is an endocrinopathy in childbearing-age females which can cause many complications, such as diabetes, obesity, and dyslipidemia. The metabolic disorders in patients with PCOS were linked to gut microbial dysbiosis. However, the correlation between the gut microbial community and dyslipidemia in PCOS remains unillustrated. Our study elucidated the different gut microbiota in patients with PCOS and dyslipidemia (PCOS.D) compared to those with only PCOS and healthy women.

**Results:**

In total, 18 patients with PCOS, 16 healthy females, and 18 patients with PCOS.D were enrolled. The 16 S rRNA sequencing in V3-V4 region was utilized for identifying the gut microbiota, which analyzes species annotation, community diversity, and community functions. Our results showed that the β diversity of gut microbiota did not differ significantly among the three groups. Regarding gut microbiota dysbiosis, patients with PCOS showed a decreased abundance of Proteobacteria, and patients with PCOS.D showed an increased abundance of Bacteroidota compared to other groups. With respect to the gut microbial imbalance at genus level, the PCOS.D group showed a higher abundance of *Clostridium_sensu_stricto_1* compared to other two groups. Furthermore, the abundances of *Faecalibacterium* and *Holdemanella* were lower in the PCOS.D than those in the PCOS group. Several genera, including *Faecalibacterium* and *Holdemanella*, were negatively correlated with the lipid profiles. *Pseudomonas* was negatively correlated with luteinizing hormone levels. Using PICRUSt analysis, the gut microbiota community functions suggested that certain metabolic pathways (e.g., amino acids, glycolysis, and lipid) were altered in PCOS.D patients as compared to those in PCOS patients.

**Conclusions:**

The gut microbiota characterizations in patients with PCOS.D differ from those in patients with PCOS and controls, and those might also be related to clinical parameters. This may have the potential to become an alternative therapy to regulate the clinical lipid levels of patients with PCOS in the future.

## Background

PCOS is an endocrine disease with a high prevalence (15%) according to the Rotterdam Consensus criteria [1]. Despite the heterogeneous symptoms and signs of PCOS, its imperative clinical manifestations include oligo-ovulation, polycystic ovaries, and androgen excess (biochemical or clinical) [2]. PCOS can exert a wide range of effects on long-term health and metabolic morbidities, such as diabetes, dyslipidemia, obesity, and coronary heart disease [3, 4]. Notably, patients with PCOS commonly have dyslipidemia, a risk factor for reproductive outcomes. Some forms of dyslipidemia, such as total cholesterol (TC), are negatively correlated with live birth in patients with PCOS [5–7]. The serum level of triglyceride (TG) is also negatively correlated with the rate of oocyte maturation [8]. Meanwhile, lipids can modulate glucose metabolism and exert side effects on early reproductive outcomes in patients with PCOS undergoing in-vitro fertilization (IVF), such as the number of retrieved oocytes [9, 10].

The gut microbiota, considered as the second genome, is an essential symbiotic partner of human cells and engages in extensive interactions with them [11]. It plays critical roles in the human host, including steroid hormone biosynthesis, immune system regulation, and metabolic health [12]. Furthermore, certain metabolites produced by the gut microbiota, such as short chain fatty acids (SCFAs), can also regulate multiple metabolic pathways [13]. Gut microbial dysbiosis might be an influencing factor in human diseases, such as obesity, PCOS, and diabetes [14, 15]. Previous studies have demonstrated a correlation between PCOS and an imbalance in the abundance in Bacteroidetes and Firmicutes [16, 17]. A recent study reported that germ-free mice transplanted with stool from patients with PCOS had elevated testosterone levels, and impaired ovarian function [18]. In addition, the gut microbial composition plays a non-negligible role in the variations in blood-lipid levels [19, 20]. The dysbiosis of gut microbiota, specifically the phyla Bacillota and Bacteroidetes, has been implicated in the regulation of lipid levels among patients with dyslipidemia [21, 22]. Therefore, aberrations in the gut microbial composition may be related to lipid metabolic disorders in patients with PCOS.

However, limited evidence has indicated a correlation between dyslipidemia in women with PCOS and gut microbial communities. In our study, we aimed to identify several important gut microbial compositions in PCOS, particularly in PCOS with dyslipidemia, and determine their association with lipid parameters and sex hormones. These findings have the potential to recommend precision therapies for PCOS patients in the future.

## Results

### Study participant characteristics

Table 1, depicts the clinical features of 52 participants. There were no significant differences in body mass index (BMI) and age between the groups. PCOS groups showed markedly higher levels of luteinizing hormone (LH), LH: follicle-stimulating hormone (LH: FSH) ratio, androstenedione, and dehydroepiandrosterone sulfate (DHEA-S) than controls. Among the three groups, no differences in parameters were observed, including progesterone, estradiol, FSH, testosterone, prolactin, homeostatic model assessment for insulin resistance (HOMA-IR), and sex hormone binding globulin (SHBG). Regarding plasma lipid parameters, patients with PCOS.D showed significantly increased levels, including TC and TG, compared with healthy controls and patients with PCOS. Furthermore, patients with PCOS.D exhibited a higher level of low-density lipoprotein cholesterol (LDL-C) than PCOS group.

**Table 1:** Clinical, endocrine, metabolic characteristics of participants in our study

Parameters	Control (*n* = 16)	PCOS (*n* = 18)	PCOS.D (*n* = 18)
Age (years)	29.81 ± 3.64	28.56 ± 4.25	27.44 ± 2.71
BMI (kg/m^2^)	22.53 ± 3.04	20.89 ± 2.85	22.70 ± 2.22
Menstrual cycle (days)	30.14 ± 1.86 ^ab^	42.69 ± 24.50 ^ac^	51.11 ± 20.91 ^bc^
Progesterone (nmol/L)	1.44 ± 1.12	1.41 ± 1.12	1.18 ± 1.15
Estradiol (pmol/L)	160.22 ± 80.98	141.81 ± 84.93	209.08 ± 182.58
LH (mIU/L)	5.33 ± 2.11^ab^	11.31 ± 6.14 ^a^	10.41 ± 4.65 ^b^
FSH (mIU/L)	6.35 ± 1.42	7.09 ± 2.40	5.94 ± 1.85
LH: FSH ratio	0.86 ± 0.35 ^ab^	1.63 ± 0.82 ^a^	1.87 ± 0.86 ^b^
Testosterone (nmol/L)	1.63 ± 1.47	2.03 ± 1.55	1.80 ± 0.69
Prolactin (ng/mL)	14.50 ± 6.11	15.73 ± 7.23	13.88 ± 5.35
Androstenedione (ng/mL)	2.50 ± 0.78 ^ab^	3.79 ± 1.75 ^a^	3.70 ± 1.45 ^b^
SHBG (nmol/L)	46.51 ± 14.89	49.38 ± 21.36	44.05 ± 30.71
DHEA-S (ug/dL)	210.80 ± 84.16 ^ab^	306.59 ± 151.08 ^a^	313.88 ± 118.91 ^b^
FPG (mmol/L)	5.23 ± 0.40	5.04 ± 0.36	5.15 ± 0.38
FINs (mU/L)	8.67 ± 2.11	7.50 ± 2.21	8.47 ± 2.27
HOMA-IR	2.02 ± 0.51	1.68 ± 0.50	1.93 ± 0.50
TC (mmol/L)	4.48 ± 0.61 ^b^	4.27 ± 0.50 ^c^	5.12 ± 0.76 ^bc^
TG (mmol/L)	0.95 ± 0.30 ^b^	0.84 ± 0.21 ^c^	2.09 ± 1.35 ^bc^
HDL-C (mmol/L)	1.43 ± 0.30	1.41 ± 0.26	1.38 ± 0.31
LDL-C (mmol/L)	2.72 ± 0.74	2.47 ± 0.69 ^c^	2.87 ± 0.91 ^c^
ALT (U/L)	14.07 ± 7.45 ^ab^	20.00 ± 11.03^a^	23.28 ± 10.23 ^b^
AST (U/L)	16.87 ± 4.67 ^ab^	19.89 ± 4.79 ^a^	21.28 ± 5.31 ^b^

The age, BMI, and menstrual cycle were obtained through questionnaires administered during the enrollment process.^a^ represent *P* < 0.05between the Control and PCOS group. ^b^ represent *P* < 0.05between the Control and PCOS.D group. ^c^ represent *P* < 0.05between the PCOS and PCOS.D group. Data are expressed as mean ± SD. Abbreviations: BMI, body mass index; LH, luteinizing hormone; FSH, follicle-stimulating hormone; SHBG, sex hormone binding globulin; DHEA-S, dehydroepiandrosterone sulfate; FPG, fasting plasma glucose; FINS, fasting plasma insulin; HOMA-IR, homeostatic model assessment for insulin resistance; TC, total cholesterol; TG, triglyceride; HDL-C, DL-C, high-density lipoprotein cholesterol; LDL-C, low-density lipoprotein cholesterol; ALT, alanine aminotransferase; AST, aspartate aminotransferase

### Gut microbial diversity among three groups

**Fig. 1:**
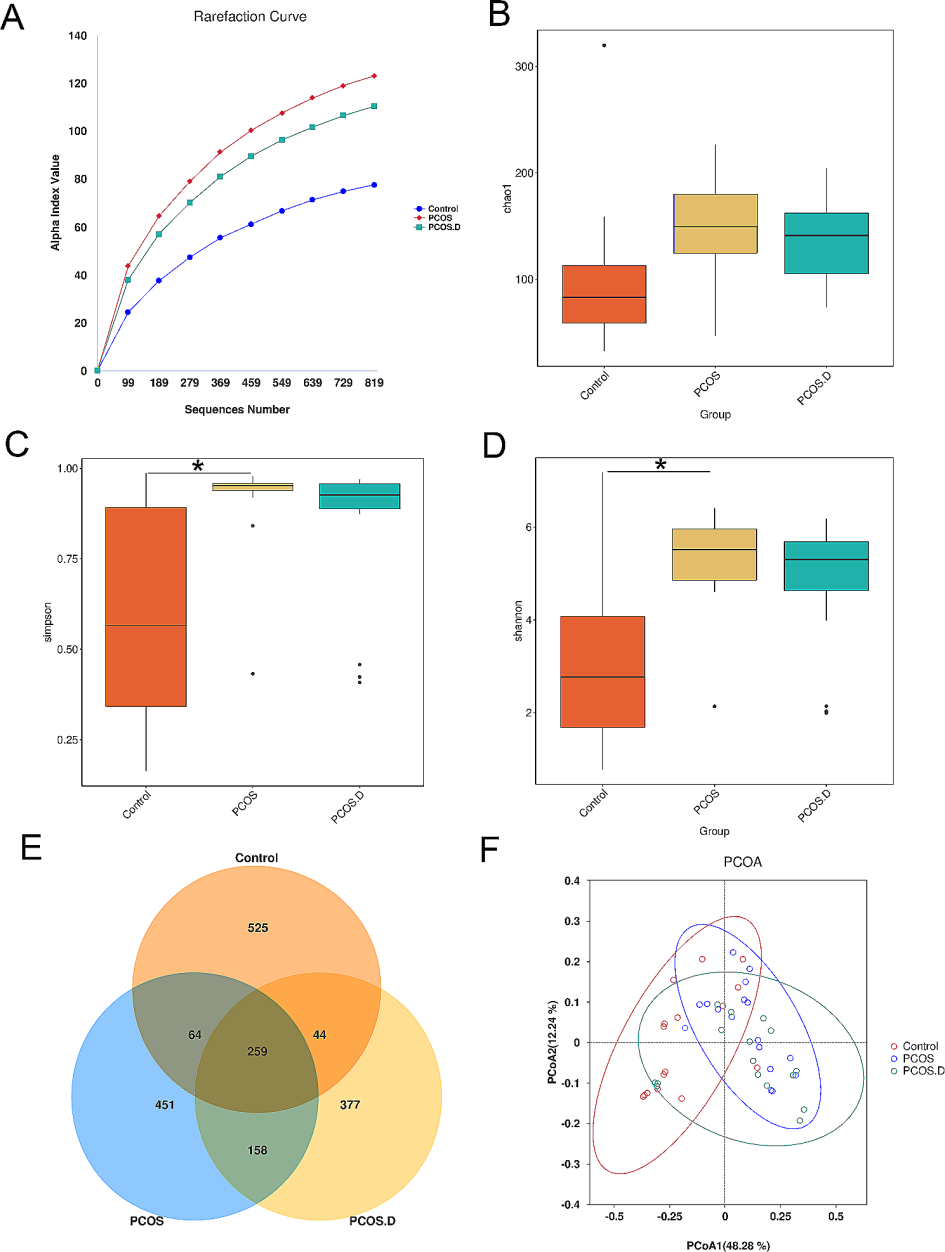
Gut microbial diversity in all participants. (**A**) The rarefaction curve based on the number of observed OTUs in the three groups. The boxplots of α diversity among the three groups in Chao1 (**B**), Simpson (**C**), and Shannon indices (**D**). (**E**) Venn diagram displaying the common and distinct OTUs among the three groups. (**F**) PCoA analysis through weighted UniFrac metric on gut microbial communities among the three groups

The α diversity of communities in the three groups was analyzed, including the number of observed operational taxonomic units (OTUs), Chao1, Simpson, as well as Shannon indices. The rarefaction curve indicated that OTU richness reached saturation in all three groups as sample sized increased (Fig. 1A). Furthermore, the α diversity of patients with PCOS was increased than that of controls. However, no significant differences between the Control and PCOS.D or between the PCOS and PCOS.D groups were observed (Fig. 1B-D). We identified common and unique genes among the three groups by comparing their gene sequences, as shown in Fig. 1E using a Venn diagram. The β diversity of the gut microbial communities was assessed according to the principal coordinate analysis (PCoA). As shown in Fig. 1F, samples from the three groups could not be completely discriminated.

### Gut microbial dysbiosis

**Fig. 2:**
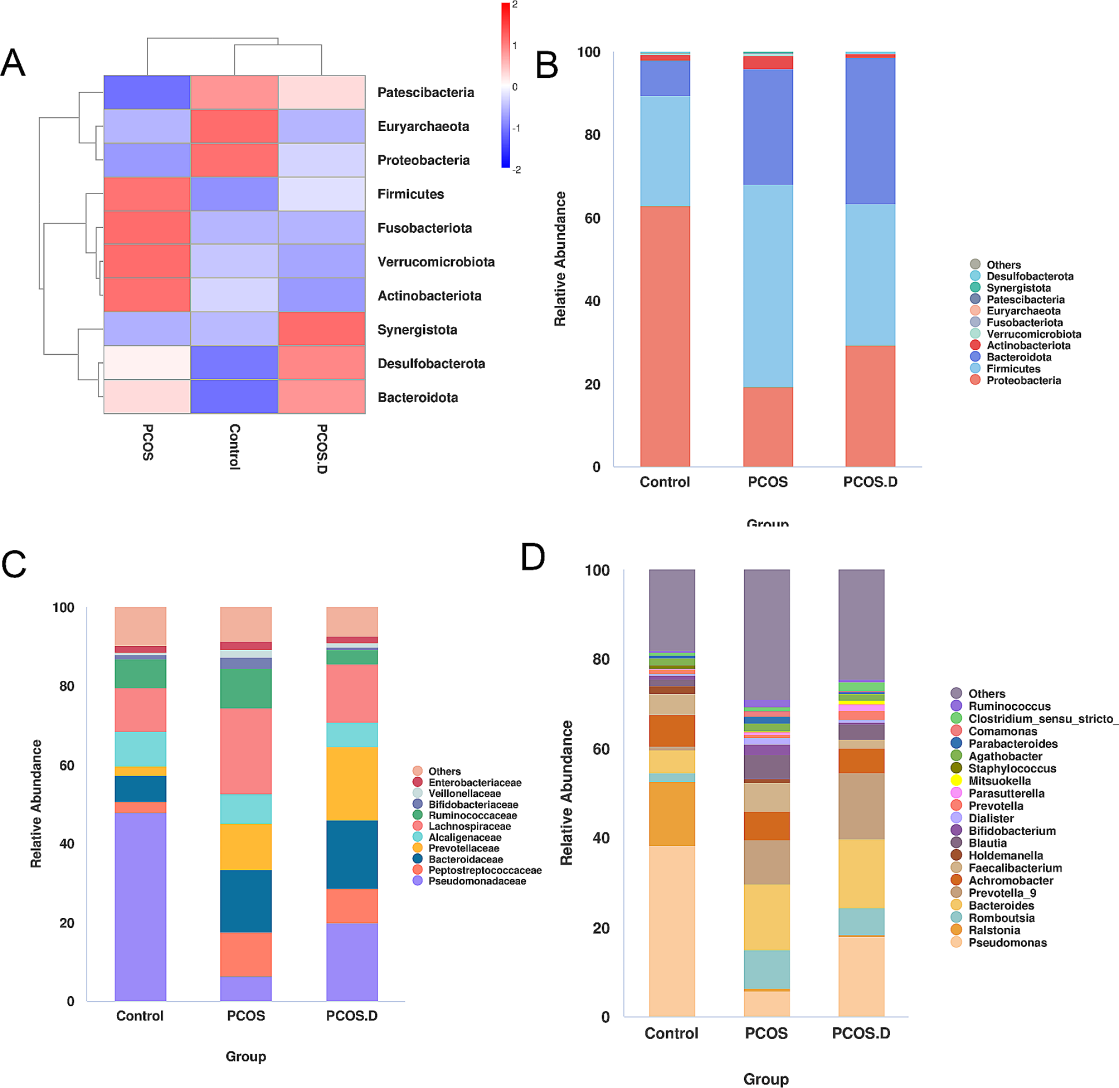
The results of gut microbiota in participants. (**A**) A phylum-level heatmap of the gut microbiota. (**B**) Gut microbiota of three groups at phylum level. (**C**) Abundance comparison of three groups at family level. (**D**) Gut microbiota of three groups at genus level

Ten bacterial phyla were identified to be different between the groups. Patients with PCOS and PCOS.D showed a notable decrease in Proteobacteria when compared to healthy women. Meanwhile, patients with PCOS showed more abundance of Firmicutes than controls. Compared to both the PCOS and Control groups, PCOS.D group exhibited a higher abundance of Bacteroidota (Fig. 2A). At the family level, the abundance of Pseudomonadaceae was decreased in the PCOS and PCOS.D groups compared to that in healthy women. In addition, PCOS.D group showed a higher abundance of Prevotellaceae than the other two groups, and PCOS group exhibited a higher level of Lachnospiraceae than the controls. The levels of Ruminococcaceae and Bifidobacteriaceae were decreased markedly in the PCOS.D than those in the PCOS group (Fig. 2B and C). In genus level, we found the abundance of *Pseudomonas* in Control group is higher compared with other groups. Patients with PCOS.D have an increased level of *Prevotella_9*, while a decreased level of *Faecalibacterium* than other two groups (Fig. 2D).

**Fig. 3:**
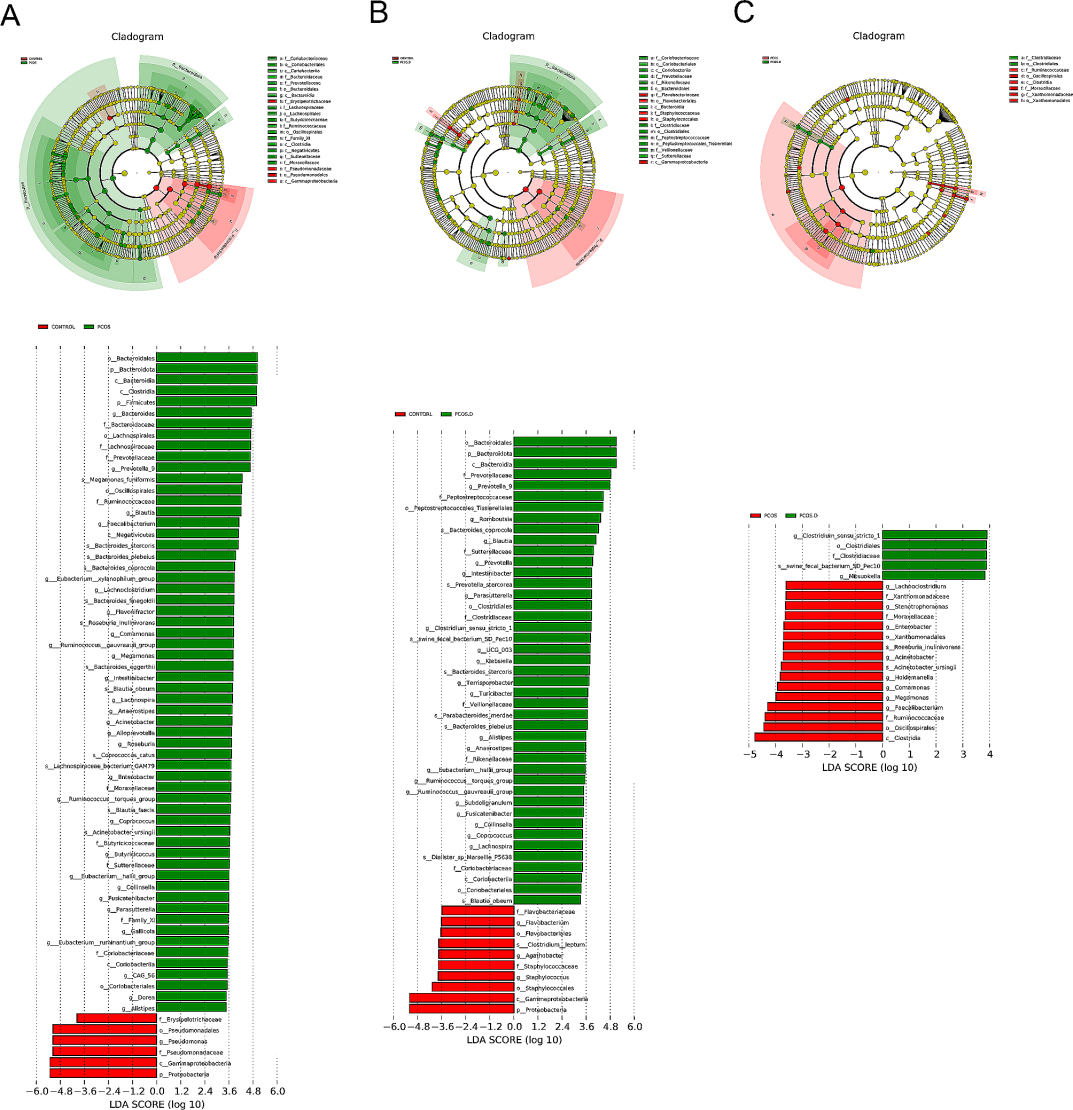
The comparison of gut bacteria at genus level among the groups. LEfSe and Cladogram analyses of significantly different classification units between Control and PCOS (**A**), Control and PCOS.D (**B**), PCOS and PCOS.D (**C**)

Next, we further analyzed the significantly different gut bacterial genera between two groups using linear discriminant analysis effect size (LEfSe) and the default of linear discriminant analysis (LDA) was three. Patients with PCOS have lower levels of *Pseudomonas* compared to healthy women, but higher levels of *Bacteroides*, *Prevotella_9*, *Blautia*, and *Faecalibacterium*(Fig. 3A). In the PCOS.D. group, the abundance of *Prevotella_9*, *Romboutsia*, and *Clostridium_sensu_stricto_1* was markedly increased when comparing with the Control group (Fig. 3B). *Faecalibacterium, Comamonas*, and *Holdemanella* were at significantly lower levels in the PCOS.D group than those in the PCOS group. Additionally, the abundance of *Clostridium_sensu_stricto_1* is higher in the PCOS.D group compared to the PCOS group (Fig. 3C).

### PICRUSt analysis

**Fig. 4:**
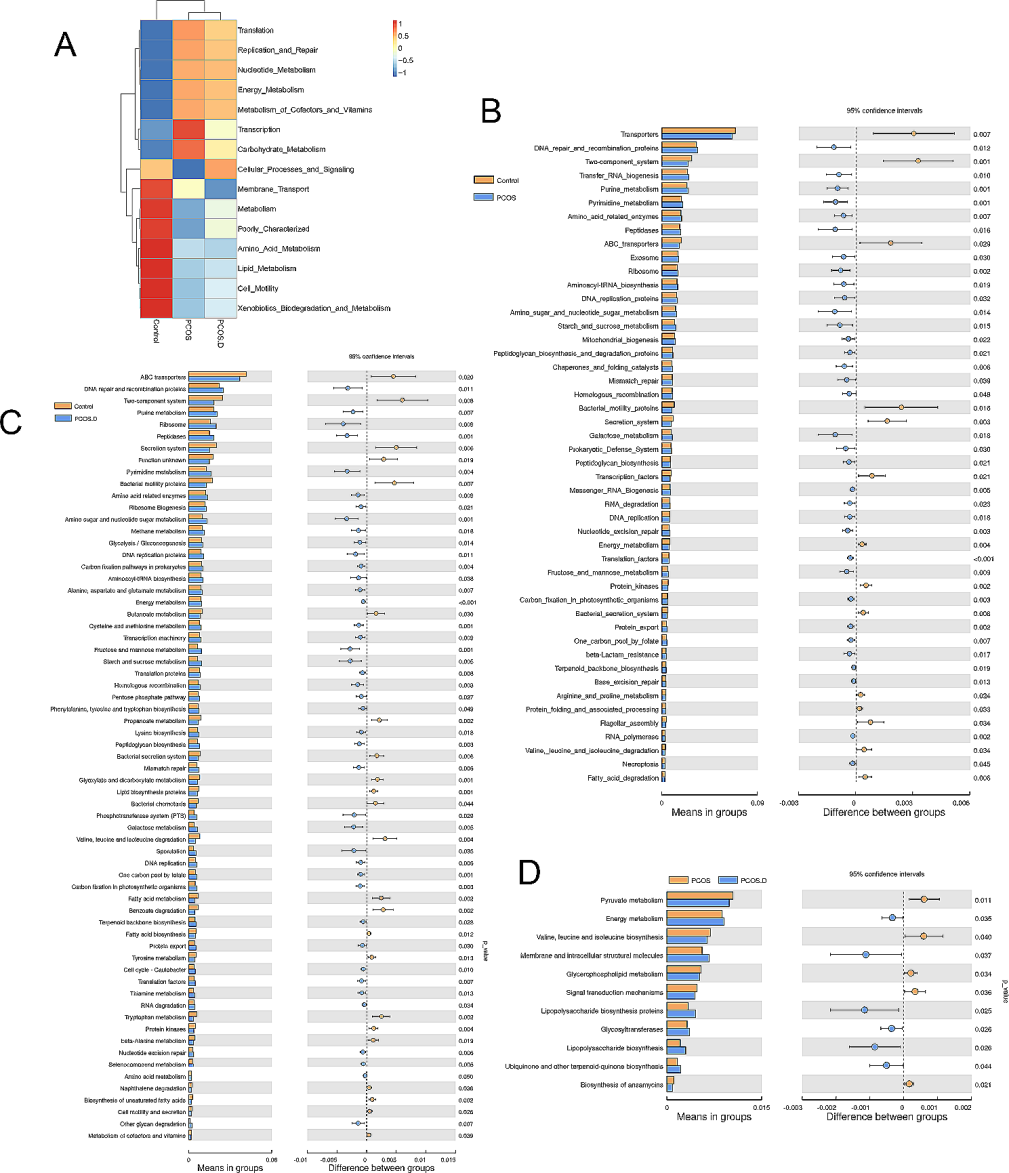
Pathway features related to gut microbial communities based on PICRUSt analysis. (**A**) The heatmap of functions in gut microbiota among the three groups. PICRUSt analysis between Control and PCOS (**B**), Control and PCOS.D (**C**), and PCOS and PCOS.D (**D**). The data was statistically significant (*P* < 0.05)

We explored the functional characterization of the gut bacteria by PICRUSt analysis. In Fig. 4A, carbohydrate metabolism was increased in patients with PCOS, but amino acid and lipid metabolism were increased in the Control group. Compared with healthy women, patients with PCOS and PCOS.D showed increased synthesis in certain amino acids (such as valine, leucine, and isoleucine) but lipid metabolism (such as lipid protein biosynthesis and fatty acid biosynthesis) was decreased (Fig. 4B and C). In addition, patients with PCOS showed a significant increase in amino acid, glycolysis and lipid metabolism compared to those patients with PCOS.D (Fig. 4D).

### Correlation analysis

**Fig. 5:**
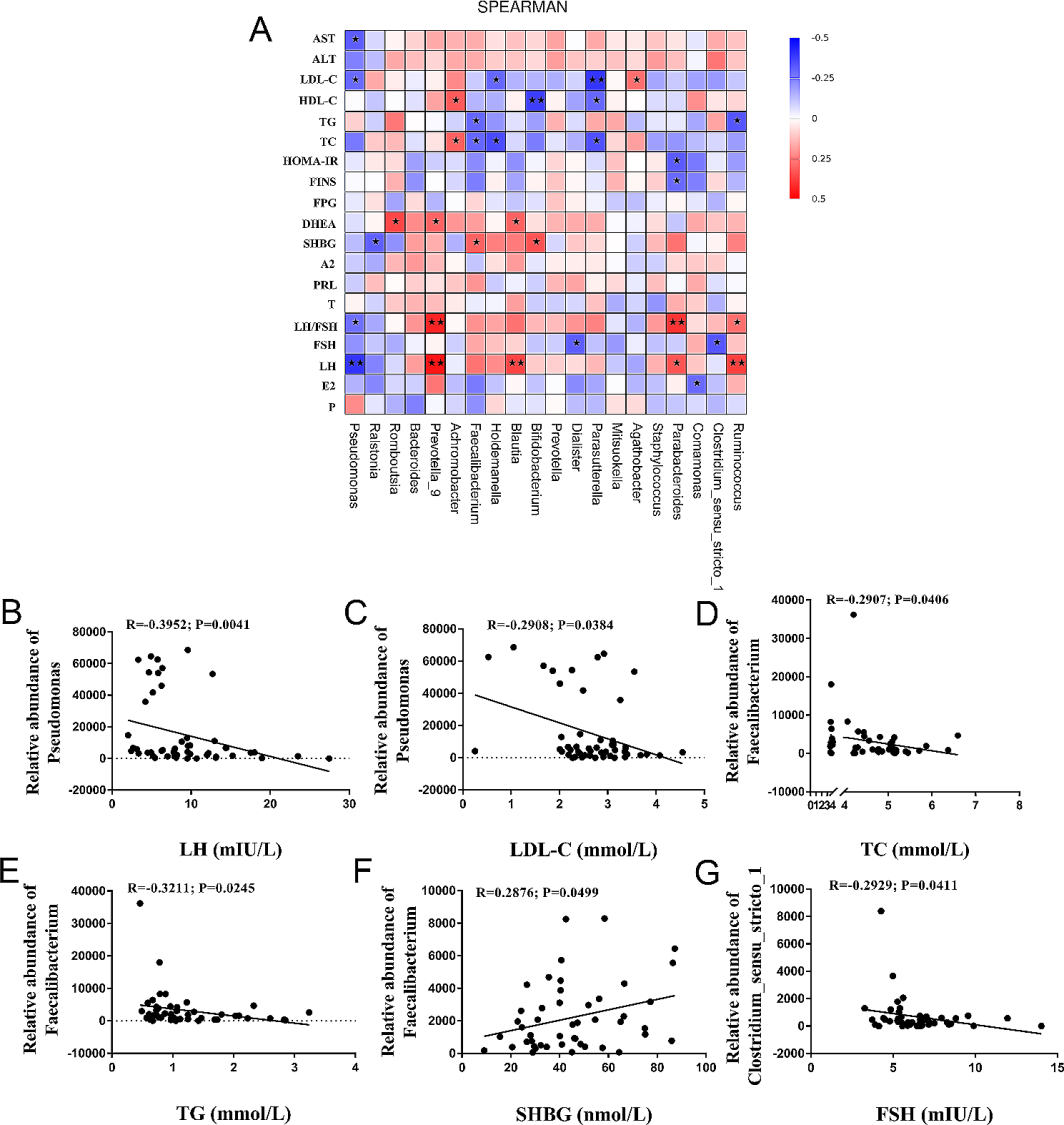
Correlation between clinical parameters in patients with PCOS and gut microbial communities. (**A**) Correlations of gut microbial genera and clinical parameters. Spot colors indicate the R value of Spearman correlation between clinical parameters and gut microbial compositions. *P < 0.05 and **P < 0.01. (**B**) The correlation between the abundance of *Pseudomonas* and the serum level of LH. (**C**) The correlation between the abundance of *Pseudomonas* and the serum level of LDL-C. (**D**) The correlation between the abundance of *Faecalibacterium* and the level of TC. (**E**) The correlation between the abundance of *Faecalibacterium* and the level of TG. (**F**) The correlation between the abundance of *Faecalibacterium* and the serum level of SHBG. (**G**) The correlation between the abundance of *Clostridium_sensu_strictio_1* and the serum level of FSH

Finally, we analyzed the correlation between clinical characteristics of the three groups and the gut microbiota. The abundance of *Prevotella_9* and *Blautia* exhibited a positive correlation with sex-hormones, including LH and DHEA levels. *Holdemanella* was negatively correlated with the level of TC and LDL-C. In addition, *Achromobacter* was positively correlated with TC and high-density lipoprotein cholesterol (HDL-C). (Fig. 5A). *Pseudomonas* displayed a negative correlation with LH and LDL-C levels (Fig. 5B and C). *Faecalibacterium* was positively correlated with SHBG, while negatively correlated with the TG and TC levels (Fig. 5D and F). Additionally, *Clostridium_sensu_stricto_1* had a significantly negative correlation with the FSH level (Fig. 5G).

## Discussion

PCOS, a heterogeneous endocrine disease in reproductive-aged females, with an estimated prevalence ranging approximately between 6 and 20%; however, this estimated may vary depending on the diagnostic criteria used [23]. Although the etiology of this disease remains unknown, PCOS is regarded as a polygenic hereditary disease that is also influenced by the environment, such as diet and lifestyle [24, 25]. In the past decade, research has established a correlation between gut microbiota and the clinical manifestations of PCOS as well as its metabolic complications [17, 26, 27]. However, the relationship of the gut microbiota and PCOS with dyslipidemia has not been investigated.

In a previous research, Torres et al. illustrated that compared with healthy women, patients with PCOS had lower α diversity and no significant clustering of unweighted UniFrac distances. Meanwhile the higher abundance of *Bacteroides* and *Porphyromonas* in PCOS patients elucidated the association between hyperandrogenism and alterations in gut microbiota [26]. However, a separate study indicated that *Akkermansia* and *Ruminococcaceae* were lower in women with obesity and PCOS, while their β diversity was significantly clustered compared with that in healthy women without obesity [16]. Numerous factors modulate the effects on the gut microbiota and host, including socioeconomic status, genes, BMI, lifestyle, and drugs [28–32]. PCOS is an intricate metabolic disease, correlated with susceptibility genes, environmental factors, and lifestyle [33, 34]. Due to the various effects of different factors on the study, we made efforts to minimize their impact by excluding women with a high BMI, taking various medicines, and having frequent diarrhea.

In our study, we found an imbalance in the gut microbiota in the patients with PCOS and those with PCOS.D. We observed a decreased abundance of *Pseudomonas* in patients with PCOS and PCOS.D compared to the controls. *Pseudomonas*, a member of the Proteobacteria phylum, is one of the most common bacteria involved in steroid degradation [35, 36]. Multiple studies have demonstrated that Proteobacteria use different pathways in steroid decomposition (such as sex hormones and cholesterol) [36]. *Pseudomonas* plays a role in degrading bile salts via candidate genes that can oxidize the corresponding aldehydes [37, 38]. In addition to investigating the mechanism of bile salt degradation in *Pseudomonas* in vitro, one study elucidated that lipase from *Pseudomonas* may hydrolyze goat blood triglycerides and cholesterol [39]. Altogether, these in vitro and in vivo results illustrate that *Pseudomonas* might be associated with lipid metabolism in patients with PCOS. Contrary to our results, a recent study on PCOS reported an increased abundance of *Pseudomonas* compared with that in healthy women, which was attributed to the sulfatase from *Pseudomonas* that could hydrolyze DHEA [40]. DHEA is then bioconverted to androstenediol [41]. Therefore, the abundance of *Pseudomonas* increased significantly in patients with PCOS. However, *Pseudomonas* also hydrolyzes testosterone to regulate steroid metabolism [42]. In the present study, we found that healthy females had an increased abundance of *Pseudomonas* compared with patients with PCOS. Meanwhile, *Pseudomonas* showed a negative correlation with serum LH and LDL-C levels. The findings suggest a potential involvement of *Pseudomonas* in patients with PCOS presenting with imbalances in sex hormone and lipid metabolism However, the mechanism between *Pseudomonas* and lipid metabolism disorders in PCOS patients should be further studied.

Furthermore, the patients with PCOS.D had a higher abundance of *Clostridium_sensu_stricto_1* than that in the other two groups. The function of this gut microbiota genus in host health is not particularly clear yet. An increased level of *Clostridium_sensu_stricto_1* is associated with metabolic disorders, such as diabetes [43], obesity [44, 45], and non-alcoholic fatty liver disease (NAFLD) according to previous studies [46].*Clostridium sensu stricto* is positively related to TG and TC levels in the patients with NAFLD [46]. *Clostridium* spp. could develop bowel inflammation [47, 48], which results in intestinal barrier dysfunction that can increase dyslipidemia [49]. These findings suggest that *Clostridium_sensu_stricto_1* has a strong association with dyslipidemia. However, interestingly, a recent study found the abundance of *Clostridium_sensu_stricto_1* had a positive correlation with HDL and a negative correlation with very-low density lipoprotein [43]. More studies are needed to reveal the role of *Clostridium_sensu_stricto_1* in PCOS.

In our study, *Faecalibacterium* and *Holdemanella* played critical roles in PCOS as compared to those in PCOS.D. *Faecalibacterium* could produce butyrate that influences carbohydrate and lipid metabolism [50, 51]. An enriched abundance of *Faecalibacterium* can ameliorate dyslipidemia and obesity [45, 52]. Similarly, *Holdemanella* genus was negatively associated with gynoid fat ratios in women [53]. Our study found that *Faecalibacterium* and *Holdemanella* were negatively correlated with TG and TC levels. Taken together, our results indicate the increasing abundance of *Faecalibacterium* and *Holdemanella*, especially *Faecalibacterium*, which might be a potential target drug development and treatment in patients with PCOS, especially those with PCOS.D.

Metabolic pathways of gut microbiota, such as amino acid and lipid metabolism, are significantly down-regulated in PCOS and PCOS.D groups identified in our results. Conversely, the gut microbiota of PCOS patients showed an upregulation in carbohydrate metabolism pathway. An imbalance in metabolites, including lipid proteins and fatty acids, may aggravate lipid metabolism dysfunction in patients with PCOS. Fatty acids can promote a pro-inflammatory response, which facilitates embryo implantation [54]. Indeed, *Pseudomonas* spp. is associated with the activation of fatty acid biosynthesis [55], which is consistent with our research. In the PCOS group, metabolic pathways of amino acids, lipid and glycolysis are increased compared with those in patients with PCOS.D. Reportedly, amino acid metabolism is able to produce SCFAs [56]. SCFAs, particularly butyrate, are important substrates for maintaining gut integrity and regulating host metabolism (e.g., glucose homeostasis and lipid metabolism) [57, 58]. *Faecalibacterium*, a key gut bacterium produces butyrate, which is negatively correlated with lipid parameters according to our results. Altogether, the reduced abundance of *Faecalibacterium* in patients with PCOS.D may be associated with lipid metabolism.

## Conclusions

In summary, our study showed that the compositions of gut microbiota were significantly different among the Control, PCOS, and PCOS.D groups. We have identified imbalanced gut compositions in patients with PCOS.D, characterized by increased levels of *Clostridium_sensu_stricto_1* and decreased abundance of *Faecalibacterium* and *Holdemanella* in PCOS.D, when compared to those in individuals with PCOS. Meanwhile, the abundance of *Faecalibacterium* was found to be significantly associated with serum lipid levels, such as TG and TC, as well as SHBG, suggesting a potential pivotal role in the pathogenesis of PCOS.D. Besides, the metabolic pathways of gut microbiota function were significantly decreased in patients with PCOS.D. However, given that multiple factors affect the host gut microbiota, the number of clinical samples should be expanded to validate our results. Further studies are warranted to reveal whether gut microbiota dysbiosis affects dyslipidemia pathogenesis in patients with PCOS. These findings could help us fully understand the gut microbial pathogenesis of PCOS and promote its personalized medicine.

## Methods

### Participants

**Fig. 6:**
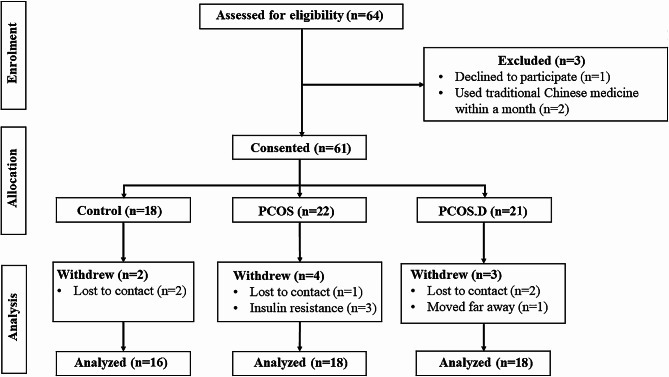
The flowchart of study participants

64 reproductive-aged women were recruited at the First Affiliated Hospital of Anhui Medical University from February 1st to December 30th 2022. However,52 participants were screened out from 64 volunteers and provided fresh stool samples. Three participants were excluded because one of them declined to participate, and the others had used traditional Chinese medicine within a month. Another two healthy participants were excluded because they failed to take part in follow-ups. One patient with PCOS was lost contact, and three patients were excluded due to insulin resistance. Additionally, another patient with PCOS.D was exclude due to relocation, and two patients were excluded for lost contact. The control group consisted of sixteen healthy females with regular menstrual periods, normal levels of sex hormones, and normal follicle morphology and count. These females underwent assisted reproduction due to “male factors.” The remaining 36 participants were stratified into two groups, PCOS (*n* = 18) and PCOS.D groups (*n* = 18), based on their lipid levels (Fig. 6). PCOS was diagnosed by the revised 2003 Rotterdam criteria; briefly, if two of the following three conditions were present: oligomenorrhea (menstrual interval > 35 d) or amenorrhea (absence of menstruation > 90 d), together with hyperandrogenism, or polycystic ovaries in ultrasound (≥ 12 antral follicles in diameter of 2–9 mm or ovary volume ≥ 10 cm^3^). Participants those had thyroid disease, elevated prolactin levels, or Cushing disease were excluded [59]. Patients with IR (HOMA-IR index ≥ 2.69) [60], as well as smoking, hypertension, diabetes, pregnancy, obesity, liver, and other serious diseases were excluded. The participants were treated without any drugs, such as antibiotics, probiotics, or metformin, for < 3 months, as evaluated by two professional physicians. All the protocols in this study were approved by the Ethics Committee of the First Affiliated Hospital of Anhui Medical University (PJ 2023-08-42).

### Clinical examination

The clinical parameters, including age, BMI (= weight[kg]/height[m]^2^) and the number of days in the menstrual cycle of all individuals were evaluated by a research assistant. Parameters (including progesterone, estradiol, LH, FSH, testosterone, prolactin, androstenedione, LDL-C, TC, TG and HDL-C) were analyzed by electrochemiluminescence immunoassay (Roche Diagnostics). The levels of TC ≥ 5.2 mmol/L, TG ≥ 1.7 mmol/L, HDL-C ≤ 1.0 mmol/L, and LDL-C ≥ 3.4 mmol/L, was defined as dyslipidemia according to the Chinese guidelines for lipid management (2023) [61].DHEA-S and SHBG were measured by chemiluminescence immunoassay (Beckman Coulter Unicel Dxl 800) Alanine aminotransferase (ALT) and aspartate aminotransferase (AST) were analyzed by a biochemical analyzer (Liaison XL, Diasorin, Saluggia, Italy).

### Sequencing

The DNA was extracted using CTAB/SDS method, followed by purity on 1% agarose gels. 16 S rRNA genes were amplified with V3-V4 primers. The PCR products underwent a denaturation and extension, then were purified by a Qiagen Gel Extraction Kit (Qiagen, Germany). After quality evaluation by a Qubit@2.0 Fluorometer (Thermo Scientific), the library was sequenced on an Illumina platform. Finally, 250 bp paired-end reads were generated.

### Bioinformatics analysis

The quality of the tags was determined by the Fastp software. The sequences were removed to obtain effective tags by Vsearch (Version 2.15.0) [62]. The QIIME2 software was used to perform species annotation. The Mothur software was used to assess α diversity (including observed OTUs, Chao 1, Simpson and Shannon index) [63]. To calculate β diversity, PCoA analysis was performed. Different community structures between groups were analyzed by ANOSIM and QIIME2 software. In addition, t-test and LEfSe analysis (LDA score threshold: three) were performed to identify the significantly different species or biomarkers [64]. Finally, functional annotation analysis was performed using PICRUSt2 software (Version 2.1.2-b).

### Statistical analysis

SPSS 24.0 (IBM SPSS, Inc., Chicago, IL, USA) was used for statistical analysis. Data was expressed as means ± standard deviations. Differences between the groups were compared by one-way ANOVA or nonparametric Mann–Whitney U test. *P* < 0.05 was considered as significantly different.

## Data Availability

The raw data used in the study are available from the corresponding author on reasonable request. The sequencing data have been submitted to China National GeneBank DataBase (accession number, CNP0004508).

## Abbreviations

PCOS: Polycystic ovary syndrome
PCOS.D: PCOS and dyslipidemia
NAFLD: Nonalcoholic fatty liver disease
BMI: Body mass index
LH: Luteinizing hormone
SHBG: Sex hormone binding globulin
HOMA-IR: Homeostatic model assessment for insulin resistance
TC: Total cholesterol
TG: Triglyceride
ALT: Alanine aminotransferase
AST: Aspartate aminotransferase
OTUs: Operational taxonomic units
PCoA: Principal coordinate analysis
ANOSIM: Analysis of similarity
LDA: Linear discriminant analysis
Lefse: Linear discriminant analysis effect size
HDL-C: High-density lipoprotein cholesterol
LDL-C: Low-density lipoprotein cholesterol SCFAs, short-chain fatty acids
DHEA-S: Dehydroepiandrosterone sulfate
IVF: In-vitro fertilization
FSH: Follicle-stimulating hormone
FPG: Fasting plasma glucose
FINS: Fasting plasma insulin
